# Direct and Indirect Effects of Management Intensity and Environmental Factors on the Functional Diversity of Lichens in Central European Forests

**DOI:** 10.3390/microorganisms9020463

**Published:** 2021-02-23

**Authors:** Steffen Boch, Hugo Saiz, Eric Allan, Peter Schall, Daniel Prati, Ernst-Detlef Schulze, Dominik Hessenmöller, Laurens B. Sparrius, Markus Fischer

**Affiliations:** 1 WSL Swiss Federal Institute for Forest, Snow and Landscape Research, Zürcherstrasse 111, CH-8903 Birmensdorf, Switzerland; 2 Institute of Plant Sciences, University of Bern, Altenbergrain 21, CH-3013 Bern, Switzerland; hugo.saizbustamante@ips.unibe.ch (H.S.); eric.allan@ips.unibe.ch (E.A.); daniel.prati@ips.unibe.ch (D.P.); markus.fischer@ips.unibe.ch (M.F.); 3 Department of Silviculture and Forest Ecology of the Temperate Zones, University of Göttingen, Büsgenweg 1, 37077 Göttingen, Germany; peter.schall@forst.uni-goettingen.de; 4 Max Planck Institute for Biogeochemistry, Hans-Knöll-Straße 10, 07745 Jena, Germany; dschulze@bgc-jena.mpg.de (E.-D.S.); dominik.hessenmoeller@forst.thueringen.de (D.H.); 5 Forstamt Schmalkalden, Thueringen Forst, Schlossberg 11, 98574 Schmalkalden, Germany; 6 BLWG, Hollandse Toren 40, 3511 BN Utrecht, The Netherlands; sparrius@blwg.nl

**Keywords:** beech forest, conifer forest, environmental filtering, forest management intensity, functional trait, habitat heterogeneity, lichen functional diversity, over-redundancy, structural equation modeling, temperate forest

## Abstract

Using 642 forest plots from three regions in Germany, we analyzed the direct and indirect effects of forest management intensity and of environmental variables on lichen functional diversity (FDis). Environmental stand variables were affected by management intensity and acted as an environmental filter: summing direct and indirect effects resulted in a negative total effect of conifer cover on FDis, and a positive total effect of deadwood cover and standing tree biomass. Management intensity had a direct positive effect on FDis, which was compensated by an indirect negative effect via reduced standing tree biomass and lichen species richness, resulting in a negative total effect on FDis and the FDis of adaptation-related traits (FDisAd). This indicates environmental filtering of management and stronger niche partitioning at a lower intensity. In contrast, management intensity had a positive total effect on the FDis of reproduction-, dispersal- and establishment-related traits (FDisRe), mainly because of the direct negative effect of species richness, indicating functional over-redundancy, i.e., most species cluster into a few over-represented functional entities. Our findings have important implications for forest management: high lichen functional diversity can be conserved by promoting old, site-typical deciduous forests with a high richness of woody species and large deadwood quantity.

## 1. Introduction

Lichens are an important component of forest ecosystems. Specifically, lichen communities constitute a substantial part of forest biodiversity [1,2,3,4] and contribute to important ecological functions [5]. However, because of lichen sensitivity to environmental changes, human-induced habitat degradation (e.g., by land-use changes) and air pollution have led to strong declines in lichen diversity during the last century [2]. Forest management is proposed to be one of the most important threat factors, responsible for a large number of threatened lichen species [2,6,7]. Despite ongoing efforts towards sustainable forest management, the promotion of biodiversity often conflicts with other goals, most prominently wood and timber production [8]. While the majority of Central European forests are managed to some extent and have management-modified dominant tree species, natural or unmanaged forests are restricted to small fragments within a production-forest landscape, covering only a very small percentage of the total forest area (<3% in Germany [9]). To meet the demands of both timber production and the development of sustainable forest ecosystems that support the conservation of lichen biodiversity, it is central to mechanistically understand how forest management affects lichen communities.

In recent years, community ecology has experienced a rise in the use of biodiversity dimensions other than taxonomic diversity. For example, measures of functional diversity (i.e., the diversity and variation of a species’ functional traits within a community trait hypervolume [10]) are increasingly used in ecological studies to explore biodiversity patterns (e.g., [11,12]). Functional traits are organismal morpho-physio-phenological characteristics that influence fitness, coexistence and the functioning of ecosystems [13]. Thus, compared with traditional taxonomic diversity indices like species richness, they can provide more general and mechanistic insights into the factors driving local differences in species composition and biodiversity dynamics, and interactions between organisms and their environment [11,13,14,15].

In contrast to vascular plants, no comprehensive and continuously maintained trait databases currently exist for lichen species, probably because of the general lack of standardized methods for measuring and calculating functional traits. Nevertheless, lichen functional traits are increasingly used in order to explore ecological processes, and measuring lichen traits for a particular set of sampled species is becoming more common (e.g., [16,17,18,19,20]). In fact, studies have recently been conducted to investigate how lichen functional diversity changes along ecological gradients (e.g., [21,22,23]) and how it is affected by forest management (e.g., [4,24,25]). However, a comprehensive assessment of how lichen functional diversity changes along a continuous gradient of forest management intensity across different regions and varying environmental conditions is still lacking.

Forest management intensity can influence lichen functional diversity in multiple ways. Increasing management intensity can have a negative direct effect on lichen functional diversity via habitat homogenization (i.e., the reduction of available colonizable substrates) and an indirect effect through the overall reduction of lichen species richness. This could be associated with the exclusion of functional traits and specialized species [26,27,28], depending on specific habitat requirements that are restricted to old forests and old trees. For example, the loss of specialized species because of reduced habitat heterogeneity should be accompanied by a reduction in functional diversity, based on traits related to the ecological adaptation of lichen species. This reduction in functional diversity can lead to functional over-redundancy (i.e., most species of a community cluster into a few over-represented functional properties), which can influence other ecosystem properties such as stability [21]. Particularly for lichen species, over-redundancy is most likely reflected by a clustering of most species into trait properties related to establishment, reproduction and dispersal. For instance, it has been demonstrated that many lichens associated with old-growth forests share specific attributes, such as requirements for specific habitat features limiting their establishment in younger stands, or morphological features, such as large diaspores limiting dispersal [4,29]. Thus, for an appropriate evaluation of lichen functional diversity, it is mandatory to consider adaptation- and performance-related traits separately in the same study. Finally, although there is a wealth of studies on the effects of forest management on lichen diversity, they mostly involved comparisons of unmanaged and managed forests at the plot scale (e.g., [30,31,32,33]), included only one study region or forest type, or were restricted to only one ecological guild such as epiphytes [34,35,36,37,38,39,40]; but see [41]. Therefore, to improve our understanding of the multiple effects of forest management on lichen functional diversity, studies considering large areas with wide management gradients are needed. Such investigations should control for potential indirect effects through species richness and should include multiple and contrasting traits.

**Figure 1:**
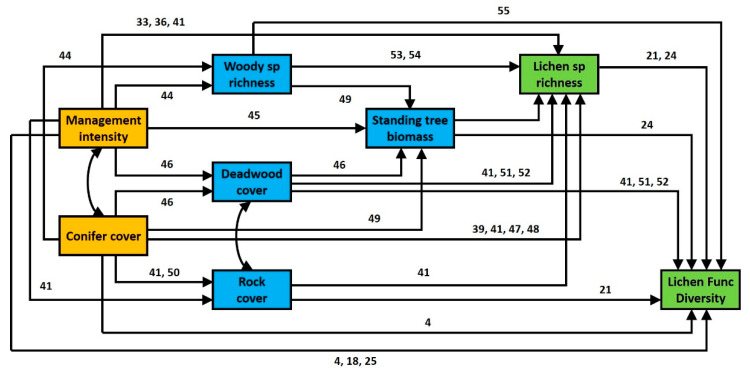
Theoretical model for the effects of forest management and conifer cover (orange boxes) on environmental variables (blue boxes) and lichen biodiversity (green boxes). Single-headed arrows indicate a direct effect of a given variable on another. Double-headed arrows indicate a mutual dependency between two variables. The numbers next to each arrow indicate references supporting the presence of that direct effect [4,18,21,24,25,33,36,39,41,44,45,46,47,48,49,50,51,52,53,54,55].

The aim of this study was to determine the importance of forest management and environmental factors, as both direct and indirect effects, for lichen functional diversity. For this, we used a large-scale dataset from the Biodiversity Exploratories project [42], which includes 642 forest plots from three regions in Germany differing in dominant tree species and management intensity. We conducted a forest inventory to quantify management intensity, recorded forest attributes and collected data on lichen biodiversity, including both taxonomic and functional diversity. We then fitted structural equation models [43] to evaluate all the potential pathways through which forest management could influence lichen functional diversity (Figure 1). We hypothesized that higher forest management intensity and a modified dominant tree species (e.g., a higher proportion of conifer trees) would act as an environmental filter, e.g., by changing stand age (indicated by standing tree biomass) and substrate availability (e.g., deadwood, rocks, number of tree and shrub species), thereby influencing lichen taxonomic diversity and ultimately reducing lichen functional diversity.

## 2. Materials and Methods

### 2.1. Study System

**Figure 2:**
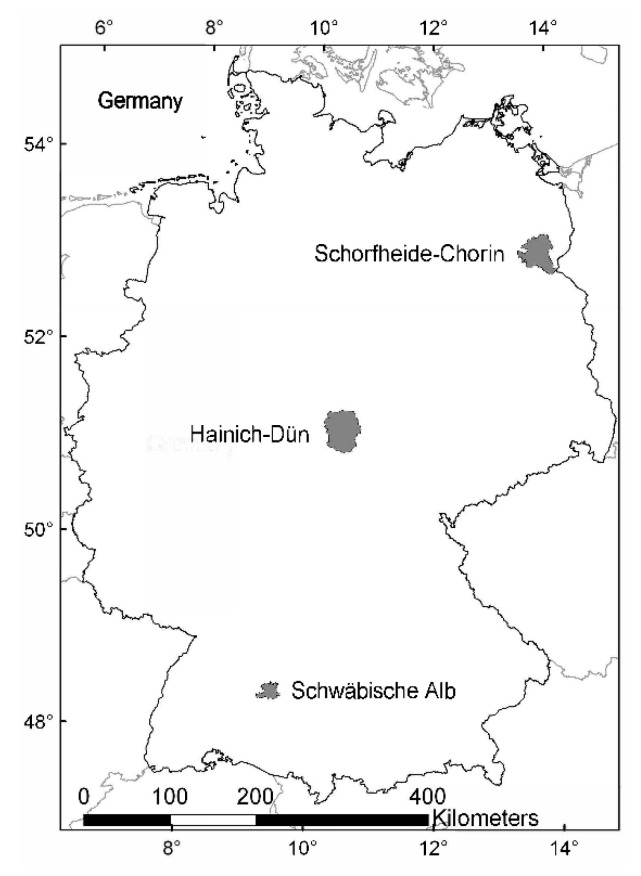
Location of the three study regions in Germany: Schorfheide-Chorin (northeast), Hainich-Dün (center) and Schwäbische Alb (southwest).

This study was conducted as part of the Biodiversity Exploratories project (www.biodiversity-exploratories.de/en/, accessed on 22 February 2021) in three regions of Germany: Schwäbische Alb (48°20′28′′–48°32′02′′ N, 09°10′49′′–09°35′54′′ E), Hainich-Dün (50°56′14′′–51°22′43′′ N, 10°10′24′′–10°46′45′′ E) and Schorfheide-Chorin (52°47′25′′–53°13′26′′ N, 13°23′27′′–14°08′53′′ E; Figure 2). The regions differ in climate, geology and topographical situation, they have different forest management systems and they have species pools that are typical for large parts of temperate Europe ([42,56]; Table 1). The UNESCO Biosphere area Schwäbische Alb (Swabian Jura) is situated in the low mountain ranges with calcareous bedrock in the southwest of Germany. Its climate has a montane character. The National Park Hainich-Dün and its surrounding areas are situated in a mid-elevation mountain range in central Germany. This region is characterized by shell limestone and Loess soils. The UNESCO Biosphere Reserve Schorfheide-Chorin is a landscape in northeastern Germany composed of post-glacial moraine. Sandy soils with variable proportions of loam predominate and the climate is sub-continental, having the lowest mean annual precipitation of the three study regions ([41,42]; for details, see Table 1).

### 2.2. Plot Selection

In a first step, a 100 m × 100 m grid was laid over the total area of each of the three study sites. More than 500 forest plots in each region were selected randomly, stratified at the intersection points of the grid, after discarding plots that fully or partially overlapped with settlements, grasslands, agricultural fields or water bodies, and plots that were intersected by roads. The forests had different management systems, from unmanaged mature deciduous forests dominated mainly by European beech (*Fagus sylvatica* L.); to age-class forests dominated by European beech, Norway spruce (*Picea abies* (L.) H.Karst.) or Scots pine (*Pinus sylvestris* L.) with different developmental stages of even-aged structure due to harvests at 80- to 120-year intervals; to uneven-aged European beech-dominated selection forests, in which single or small groups of trees were harvested selectively [42,56]. From these plots, we randomly selected 642 plots (158 in the Schwäbische Alb, 175 in Hainich-Dün and 309 in Schorfheide-Chorin) for this study. As these plots covered all management types and intensities in each region, we consider our plot sample unbiased for studying differences in forest management.

### 2.3. Forest Management Intensity

In each plot, a forest inventory was conducted on a circular 500-m^2^ plot (radius 12.62 m) that was concentric with our plot to assess the stand characteristics of forests, such as the diameter at breast height (DBH) and height of each tree using an ultrasonic tree height meter (Vertex III Forester, Haglöf, Langsele, Sweden), as well as the number of trees (>7 cm DBH). We then calculated standing tree biomass (m^3^/ha) using the height and DBH of each occurring tree, accounting for tree species-specific trunk shapes (for details see [49]). In addition, the basal area (BA) of each plot was calculated. We then quantified forest management intensity (SMId sensu [45]) by relating the basal area to the carrying capacity (BAcc) of the forests. This measure thus accounts for the deviation of the actual stocking from a fully stocked mature forest (=1 – BA × BAcc^−1^), which has been modified by harvests and thinning. Basal area carrying capacity was quantified for each tree species using the 95% quantile of observed values for European beech-dominated forests as a reference (45 m^2^ ha^−1^). The carrying capacity of other tree species was estimated relative to European beech based on yield tables (spruce and fir: 63 m^2^ ha^−1^; pine and larch: 51 m^2^ ha^−1^; Douglas-fir: 69 m^2^ ha^−1^; oak and other broadleaved species: 36 m^2^ ha^−1^). In mixed stands, forest management intensity was quantified relative to the current tree species composition (=1 – Σ BAi × BAcc,i^−1^, with i representing tree species).

### 2.4. Vegetation Data and Environmental Forest Variables

During 2007 and 2008, the first author recorded all lichen species in an area of 20 m × 20 m in each of the 642 plots (always positioned at the intersection points of the 100 m × 100 m grid from which the 500 forest plots had been selected), including saxicolous species on rocks and stones, lignicolous species on deadwood, terricolous species on soil, and epiphytes on the bark of woody vascular plant species (up to 2.5 m height on tree trunks and the branches of shrubs, likely underestimating the total lichen species richness because species restricted to tree crowns were not assessed [57,58]). Most lichen species were identified in the field. Critical specimens were collected and identified in the laboratory. The nomenclature of lichen species follows Scholz ([59]; see Table S2 for the full species list). We further recorded all shrub (woody species 0–5 m in height, excluding seedlings) and tree species (>5 m in height) in each plot and estimated the percentage cover of each species. We then calculated the number of woody species (i.e., shrubs and trees) as a measure of substrate availability for epiphytic lichen species. In addition, we calculated the cumulative cover of woody species, as well as the proportion of conifers, as a continuous measure instead of using coniferous, deciduous and mixed forests as categories. Furthermore, we visually estimated the percentage of ground covered by deadwood and rocks for a measure of substrate availability for lignicolous and saxicolous lichen species.

### 2.5. Functional Trait Data and Functional Diversity Calculations

For the total of 201 lichen taxa found, we extracted information for a set of 15 lichen functional traits (see Table S1 in the Supplementary Material) from identification keys, other common literature sources [16,60,61] and databases [62,63]. As additional functional traits, we used ecological indicator values for light, temperature, moisture, acidity, eutrophication and continentality of lichen species [64], as well as a temperature index [65]. The aim of ecological indicator values (EIV) is to quantify the environmental niche of a particular species on an ordinal scale (1 is the lowest and 9 is the highest value in the case of [64]). These values are not based on measurements but on expert knowledge. However, as they often reflect site conditions better than exact point measurements [66], EIVs are often used in ecological studies, either averaged per plot to describe the environmental conditions of a site, e.g., to visualize temporal changes of ecological conditions in vegetation plots (e.g., [67,68]), or as additional functional traits in studies exploring the functional diversity patterns of lichens [4,18].

We then assigned each trait to a group, either reflecting the ecological adaptation (growth form, photobiont type, secondary metabolite production, ecological indicator values and temperature index; for details, see Supplementary Table S1) or the reproduction, dispersal and establishment (vegetative diaspore size, conidia propagules, fungal propagules, ascomata area, spore septation, spore shape and spore volume; for details, see Supplementary Table S1) of lichen species. Finally, for each group, we selected the five traits that showed the lowest correlation among them (|*r*| < 0.35; Supplementary Table S2). Thus, the final trait selection for functional diversity analysis included growth form, photobiont type, EIV light, EIV reaction and EIV moisture for ecological adaptation; and vegetative diaspore size, ascomata area, spore septation, spore shape and spore volume for reproduction, dispersal and establishment.

For each plot, we calculated functional diversity using the functional dispersion index (FDis, the mean distance in multidimensional trait space of individual species to the centroid of all species [10]). Functional dispersion allows us to calculate diversity for multiple traits based on inter-trait distance measures; thus, communities composed of species with similar trait values will have a low FDis, while those with species with differentiated traits will have a high FDis. Specifically, we calculated three FDis metrics per plot: FDisAd, which accounted for the functional diversity associated with ecological adaptation traits; FDisRe, which accounted for the functional diversity associated with reproduction, dispersal and establishment traits; and FDisTotal, for the functional diversity considering all functional traits together. Each FDis measure was calculated with the FD package in R [69], using the Gower dissimilarity method because it calculates distance matrices by simultaneously integrating continuous and categorical variables [70]. As comparing FDis between sites is sensitive to differences in species richness [71], we additionally applied a null model where we randomized within-plot lichen identity by selecting the same number of lichen species from the regional pool (i.e., all lichen species present in the study system) used to calculate FDis metrics. Specifically, we ran 100 simulations per plot and calculated the expected functional dispersion for each index as the average of the simulated functional dispersion. Finally, for each plot, we defined functional dispersion as the difference between the observed and the expected values.

### 2.6. Data Analysis

We used structural equation modeling (SEM) [43], which is a powerful statistical tool to separate direct and indirect effects in well-replicated comparative studies (e.g., [72]). This approach is increasingly used in ecological studies but has only very rarely been used to explore patterns of lichen diversity and composition in forests (but see [53,55,73]). Specifically, we evaluated the effects of forest management intensity (SMId) and conifer cover, as well as environmental variables (rock and deadwood cover, standing tree biomass, woody plant species richness) and lichen species richness on lichen functional diversity. In our models, management intensity and conifer cover had a direct effect on all other variables, environmental variables had a direct effect on lichen species richness and functional diversity, and lichen species richness had a direct effect on lichen functional diversity. In addition, we included the correlation between management intensity and conifer cover, and the correlation between deadwood and rock cover in our models. We ran three SEMs using the same model structure but changing the functional diversity measure: one for FDisAd, one for FDisRe and one for FDisTotal. Importantly, the SMId measure does not account for differences in tree species composition; thus, the different management categories (unmanaged, age class and continuous forest) needed to be considered in the analysis. However, as management type did not have a significant effect on any FDis index (Supplementary Figures S1–S3), we did not include management type in the models and we focused on the effect of SMId. In all models, variables were transformed to meet normality assumptions and scaled before the analysis, and we worked with the residuals of the data after removing the effect of region (Schwäbische Alb, Hainich-Dün, Schorfheide-Chorin) and survey year (2007, 2008). We conducted all analyses using R 3.5.3 [74] and calculated all SEMs with the lavaan package in R [75].

## 3. Results

### 3.1. General Effects of Forest Management Intensity and Ecosystem Components on Lichen Functional Diversity

**Figure 3:**
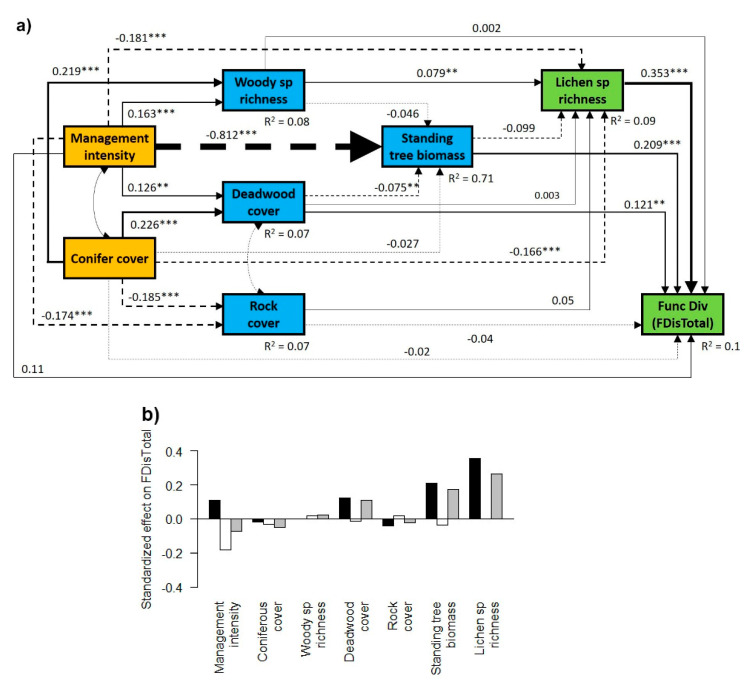
Effects of forest management and environmental variables on the functional diversity index considering all functional traits together (FDisTotal). (**a**) Structural equation model depicting the direct and indirect effects of forest management and conifer cover (orange boxes) on environmental variables (blue boxes) and lichen biodiversity (green boxes). Functional diversity was calculated for all traits (FDisTotal). Single-headed arrows indicate a direct effect of a given variable on another. Double-headed arrows indicate a mutual dependency between two variables. The numbers adjacent to the arrows show standardized path coefficients; the widths of the lines are proportional to the size of the path coefficients. Solid lines indicate positive and dashed lines negative relationships. Asterisks next to the path coefficients indicate *p*-values: ***, *p* < 0.001; **, *p* < 0.01; no asterisk, *p* ≥ 0.05 (n.s.). The dashed arrows show co-variances between factors. *R*^2^ values denote the proportion of variance explained for the endogenous variables. (**b**) Direct effects (black bars), indirect effects (white bars) and standardized total effects (direct × indirect effect; grey bars) of all factors on FDisTotal, derived from the structural equation models.

**Figure 4:**
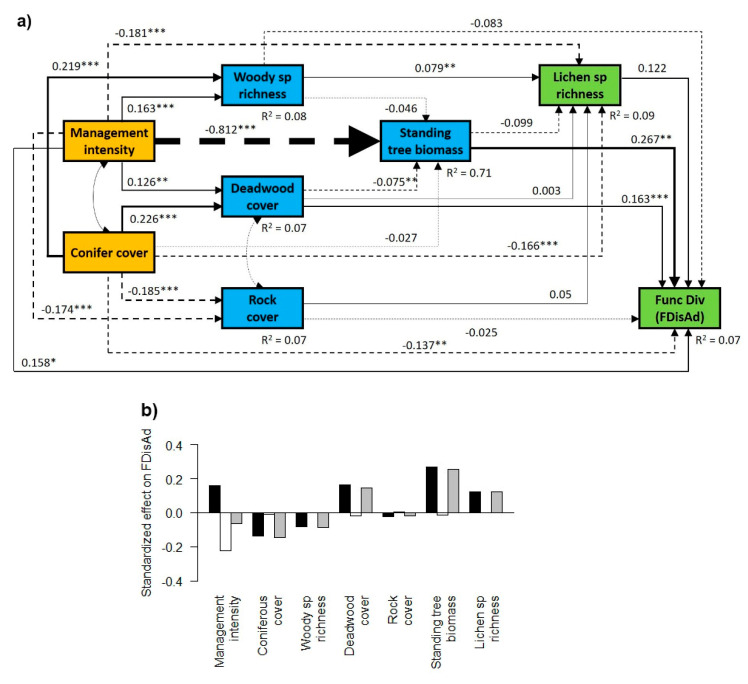
Effects of forest management and environmental variables on the functional diversity index based on adaptation-related traits (FDisAd). (**a**) Structural equation model depicting the direct and indirect effects of forest management and conifer cover (orange boxes) on environmental variables (blue boxes) and lichen biodiversity (green boxes). Functional diversity was calculated for adaptation-related traits (FDisAd). Single-headed arrows indicate a direct effect of a given variable on another. Double-headed arrows indicate a mutual dependency between two variables. Numbers adjacent to the arrows show standardized path coefficients; the widths of the lines are proportional to the size of the path coefficients. Solid lines indicate positive and dashed lines negative relationships. Asterisks next to the path coefficients indicate *p*-values: ***, *p* < 0.001; **, *p* < 0.01; *, *p* < 0.05; no asterisk, *p* ≥ 0.05 (n.s.). The dashed arrows show co-variances between factors. *R*^2^ values denote the proportion of variance explained for the endogenous variables. (**b**) Direct effects (black bars), indirect effects (white bars) and standardized total effects (direct × indirect effect; grey bars) of all factors on FDisAd, derived from the structural equation models.

**Figure 5:**
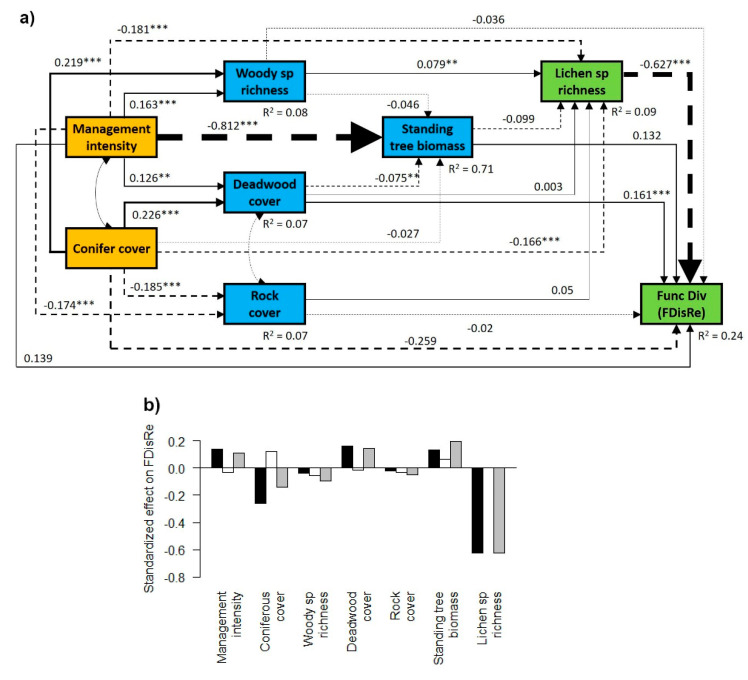
Effects of forest management and environmental variables on the functional diversity index based on reproduction-, dispersal- and establishment-related traits (FDisRe). (**a**) Structural equation model depicting the direct and indirect effects of forest management and conifer cover (orange boxes) on environmental variables (blue boxes) and lichen biodiversity (green boxes). Functional diversity was calculated for reproduction-, dispersal- and establishment-related traits (FDisRe). Single-headed arrows indicate a direct effect of a given variable on another. Double-headed arrows indicate a mutual dependency between two variables. Numbers adjacent to the arrows show standardized path coefficients; the widths of the lines are proportional to the size of the path coefficients. Solid lines indicate positive and dashed lines negative relationships. Asterisks next to the path coefficients indicate *p*-values: ***, *p* < 0.001; **, *p* < 0.01; no asterisk, *p* ≥ 0.05 (n.s.). The dashed arrows show co-variances between factors. *R*^2^ values denote the proportion of variance explained for the endogenous variables. (**b**) Direct effects (black bars), indirect effects (white bars) and standardized total effects (direct × indirect effect; grey bars) of all factors on FDisRe, derived from the structural equation models.

In general, management intensity (SMId) and conifer cover had a direct positive effect on the woody species richness and deadwood cover, and a direct negative effect on standing tree biomass, rock cover and lichen species richness (Figure 3a, Figure 4a and Figure 5a). However, they showed contrasting effects on lichen functional diversity, with a direct positive effect of management intensity and a negative direct effect of conifer cover (Figure 3a, Figure 4a and Figure 5a), although the significance of these effects depended on the trait considered (Figure 3a, Figure 4a and Figure 5a). When summing the direct and indirect effects, we found a general negative total effect of conifer cover and a positive total effect of deadwood cover and of standing tree biomass, while the effect of rock cover on lichen functional diversity was negligible (Figure 3b, Figure 4b and Figure 5b).

### 3.2. Contrasting Findings for FDisTotal, FDisAd and FDisRe

Analyzing functional diversity based on adaptation-related (FDisAd) and reproduction-, dispersal- and establishment-related (FDisRe) traits separately, we found contrasting responses (Figure 3, Figure 4 and Figure 5). While the indirect negative effect of management intensity on FDisTotal and FDisAd, via reduced standing tree biomass and lichen species richness, was stronger than the positive one, resulting in a negative total effect (Figure 3 and Figure 4), management intensity had a positive indirect and total effect on FDisRe (Figure 5b). This indirect positive effect was mainly driven by the strong negative effect of species richness and the weak and non-significant effect of standing tree biomass on FDisRe (Figure 5a), which was in contrast to the positive effect of species richness on FDisTotal and the positive effect of standing tree biomass on FDisTotal and FDisAd (Figure 3a and Figure 4a).

## 4. Discussion

In this study, we used structural equation modeling to more mechanistically explore the complex responses of the taxonomic and functional diversity of lichens to forest-management-related changes in environmental variables. Our results showed that forest management influenced lichen functional diversity through multiple paths, and particularly that indirect effects could be more important than direct ones. In addition, we found that functional entities respond differently to management, with contrasting responses for traits related to adaptation compared with those related to reproduction, dispersal and establishment. Altogether, our study highlights the necessity to consider multiple ecosystem components if the aim is to achieve sustainable management for temperate forests.

### 4.1. Direct Effects of Management Intensity on Environmental Variables and Related Effects on Lichen Species Richness

We found pronounced effects of management intensity on environmental variables, such as the availability of substrates colonizable by lichens, which ultimately cascaded to lichen species richness. We cannot definitively explain the direct negative effect of management intensity on rock cover. To our knowledge, rocks are not actively removed during management interventions. It could be that, due to canopy openings caused by forest management, terricolous bryophytes are promoted [50] and cover the surface of rocks, which are then no longer visible and go undetected in surveys. In contrast, the positive effect of management intensity on deadwood cover can be directly attributed to management: the retention of logging debris and stumps in recently managed stands leads to larger deadwood volumes in managed than in unmanaged stands, and in coniferous stands, which were, on average, more frequently harvested, than in deciduous forest stands [46,50,76]. In addition, management-related disturbances can promote the richness of vascular plant species [44], which could explain the positive effect of management intensity on the richness of woody species found in this study, which, in turn, positively affected the number of lichen species. There are various studies reporting a positive association between lichen and tree species richness (e.g., [53]), which can be attributed to the different substrate and microhabitat provision of different tree and shrub species [54]. Such specific physical and chemical properties additionally often change on the individual tree level, either over time or along vertical gradients from the base to the crown, leading to vertically changing lichen species assemblages and to a higher total lichen richness [57,77]. Finally, the observed negative relationship of forest management with standing tree biomass was expected, since standing biomass decreases with a decreasing natural stocking rate [45]. However, we preferred to keep SMId and standing tree biomass together in the model to explore the potential direct and indirect effects of management on lichen biodiversity.

In our study, an increase in forest management intensity directly reduced lichen species richness. Likewise, Boch et al. [41] found more lichen species in older stands with high values of standing tree biomass. The authors attributed their findings to the preference of specialized species for older stands and trees, which provide more microhabitats than younger ones, and to the longer colonization time associated with these stands [37,38]. In addition, forest management affects tree canopy density, with low intensities resulting in microclimate conditions more typical for forests, with less variation in temperature [78], light availability [79] and humidity [80]. Such conditions are considered to be favorable for epiphytic species and particularly for forest specialists [81,82,83,84]. On the other hand, the negative effect of coniferous cover on lichen species richness is in line with previous studies in which lower overall lichen species richness or the richness of threatened and epiphytic lichen species was reported in conifer plantations than in native deciduous stands [41,47]. This finding can be attributed to the disrupted ecological continuity and altered environmental conditions and microhabitat variables associated with previous clear cuts such as light and humidity conditions, and the replacement of site-characteristic forests with even-aged coniferous plantations [85,86,87]. In addition, coniferous trees are suggested to provide less favorable conditions for epiphytic species than deciduous trees, e.g., because of differing physico-chemical bark properties [85,88,89]. Altogether, our findings show the complex effects of management intensity on multiple forest components and environmental variables, and they highlight the importance of conducting integrative studies to fully understand lichen community responses to human activities.

### 4.2. Direct and Indirect Effect of Management Intensity and Environmental Variables on Lichen Functional Diversity

We found a direct positive effect of forest management intensity on lichen functional diversity. This finding partly contradicts our hypothesis of the negative effect of higher management intensity on lichen functional diversity via reduced habitat heterogeneity. The positive effect of higher management intensity on substrate availability (i.e., deadwood cover and the woody plant species richness), which also has a positive effect on functional diversity, suggests that management-related disturbances can increase habitat heterogeneity, at least to a certain degree. The observed positive relationship between deadwood cover and lichen species richness is in line with findings from previous studies [41,51,90] and can be attributed to niche differentiation because of microhabitat and substrate availability. This result highlights the need to actively enhance deadwood quantity, as well as the diversity of deadwood decay stages and types (e.g., standing deadwood and deadwood on the ground), especially in older stands that have not yet reached the degeneration phase and therefore have limited deadwood amounts. Finally, we found a direct negative effect of coniferous tree cover on lichen species richness and functional diversity, resulting in a total negative effect on functional diversity (Figure 3). This corresponds to the findings of Łubek et al. [4], who studied lichen functional diversity in different forest types of the Białowieża National Park in Eastern Poland. The authors found the lowest functional diversity in coniferous forests, which had low niche heterogeneity and environmental filtering caused by harsh environmental conditions, in contrast to high functional diversity in deciduous stands with stronger niche partitioning.

Importantly, all our investigated unmanaged stands had formerly been managed and still showed signs of human activity, such as a fairly even-aged structure and dense canopy cover. Thus, they probably have not yet reached the degeneration phase with a natural level of habitat heterogeneity and the fully developed features of old-growth forests, such as large amounts of deadwood and over-mature trees [40,91]. In addition, the older stands with low management intensity were mostly dominated by European beech, which is characterized by a dense canopy cover and low light conditions [44,87]. These particular environmental conditions might promote a certain set of functional entities of lichens (e.g., mainly crustose species, species with low light EIV, lichen species with *Trentepohlia* algae and cyanobacteria, or a particular set of secondary metabolites [64,92,93,94]), resulting in a lower functional diversity of lichens. This corresponds well with our findings of enhanced functional diversity of adaptation-related traits (FDisAd) with higher forest management intensity. Likewise, Lelli et al. [25] investigated the diversity patterns of several taxa in forests dominated by European beech in Denmark and also found increased the functional diversity of lichen resulting from management-related canopy gaps, promoting more diverse lichen growth forms and lichen species with vegetative reproductive strategies. In particular foliose and fruticose lichen species might profit from increased light conditions [95,96,97]. Moreover, increasing management intensity and higher light availability can improve growing conditions for lichen species with trebouxiod photobionts [16]. However, in our case, the positive effect of management intensity on the functional diversity of adaptation-related traits (FDisAd) was compensated by the strong indirect negative effects via reduced standing tree biomass and lichen species richness, resulting in a negative total effect. These findings highlight the use of structural equation modeling to separate direct from indirect effects, enabling more mechanistic explorations of the complex responses of taxonomic and functional diversity to management-related changes in environmental conditions. Neglecting such important indirect effects of forest management on lichen biodiversity might even result in misleading conclusions and management recommendations.

Although we investigated old and unmanaged stands with a high standing biomass of trees that had not yet reached the degeneration phase, they still harbored a high functional diversity of lichens. This suggests that these stands developed special microhabitats during succession, leading to niche differentiation and thereby promoting overall species richness and the coexistence of contrasting strategies, indicated by higher functional diversity [98]. For a subset of the plots investigated in our study, Schall et al. [87] demonstrated that unmanaged forests have already evolved old-growth forest attributes, such as higher richness and a greater abundance of microhabitats compared with even-aged managed stands. This underlines our findings and confirms the hypothesis that low forest management intensity has positive effects on lichen functional diversity via stand continuity, habitat heterogeneity and increased species richness. In line with other studies highlighting the importance of old trees and stands for lichen species richness [37,38,41], our findings point out the high value of old and unmanaged stands for the conservation of lichen functional diversity.

Interestingly, and contrary to our expectations, we found an overall negative effect of lichen species richness on the functional diversity of reproduction-, dispersal- and establishment-related traits (FDisRe). As we used diversity-corrected functional diversity values in our models, this finding reflects the true lichen diversity effect on functional diversity. In this particular case, high-richness lichen communities, which are related to old, deciduous stands with low management intensity and high volumes of standing tree biomass, show a low diversity of functions related to reproduction, dispersal and establishment in comparison to communities with low richness. This indicates functional over-redundancy, leading to the selection of a particular set of shared functional traits related to old-forest conditions (i.e., most species of a community cluster into a few over-represented functional properties). This finding corresponds to those of Giordani et al. [21], who investigated the functional diversity of epiphytic and saxicolous lichen communities on the island of Sardinia and found that particular abiotic conditions drove the process of trait selection, leading to functional over-redundancy in species-rich lichen communities. Likewise, Bässler et al. [24] studied lichen functional diversity along an elevation gradient in the Bohemian Forest in Germany and found increasing species richness and decreasing functional diversity of lichens with increasing elevation, reflecting trait clustering and the selection for particular shared traits. Similar to our findings, the reduction of functional diversity could be attributed mainly to reproduction-, dispersal- and establishment-related traits. Our findings of functional over-redundancy might be driven by the loss of a certain set of well-dispersed pioneer species (e.g., those with small spores [99,100]) and generalist species during succession on the one hand. On the other hand, our findings indicate that many lichens related to older stands share specific attributes, such as requirements for specific habitat features. Features of old trees include pronounced bark textures including crevices [35,101] and rot holes [102], which provide a range of microhabitats. The fact that many lichen species associated with such features can only establish on older trees, limits their establishment in younger stands [29,103]. In addition, many lichen species are excluded from young stands because of certain morphological features limiting their dispersal, such as large vegetative diaspores [4,29].

### 4.3. Implications for Forest Management

Our findings of a positive direct and a strong negative indirect effect of higher management intensity, in combination with the negative effect of coniferous tree cover and the positive effect of deadwood cover, on lichen functional diversity, as well as the positive effect of woody plant species richness on lichen species richness (which increases overall functional diversity), have important implications for forest management. To meet the demands of both timber production and the conservation of high overall functional diversity of lichen, representing the highest scores of functional variation, we emphasize the importance of promoting stands with a high standing biomass of site-typical deciduous species and over-mature trees [41,104,105], thus enhancing the richness of woody plant species [53,73] and the deadwood quantity in older stands [41,47,51]. We additionally suggest the implementation of a mixture of different management systems, including both unmanaged and managed forests. Thus, our recommendations partly contradict the ones of Schall et al. [56], who investigated the response of γ-multidiversity to different forest management systems at the landscape scale and emphasized that γ-multidiversity can be maximized by using a single management system, i.e., traditionally managed even-aged shelterwood forests with different developmental phases. In line with our recommendations, however, the authors—who also reported the highest lichen alpha diversity in unmanaged forests—highlighted the general importance of including a certain share of other management systems, such as uneven-aged and unmanaged forests for the diversity of some taxa. Our observation of low functional diversity for reproductive traits in species-rich communities also suggests that combining various management systems and forest types at the landscape scale could maximize functional diversity. On the other hand, this finding highlights the particular importance of preserving mature stands for the conservation of a specialized set of selected traits, which might be particularly vulnerable to environmental and management changes.

## 5. Conclusions

Our findings demonstrate that analyzing the response of functional diversity to forest management intensity makes it possible to consider a dimension of biodiversity beyond pure species richness. In contrast to pure species richness analyses, which might neglect important ecosystem functions provided by a particular set of functional traits, analyses of functional diversity can provide insight into local differences in species communities and biodiversity dynamics, and interactions between organisms and their environment. Further, our study illustrates that structural equation modeling enables more mechanistic explorations of the complex responses of taxonomic and functional diversity to changes in environmental variables in response to forest management practices. Importantly, neglecting the indirect effects of forest management on lichen biodiversity through habitat changes can lead to incomplete conclusions and compromise the sustainable development of forests, as these effects are sometimes more important than the direct effects. Considering functional diversity measures, especially in combination with structural equation modeling, could therefore could therefore improve management recommendations based on the analysis of species richness patterns.

## Figures and Tables

**Table 1:** Main geographic, climatic and habitat characteristics of the three study regions, as well as mean, minimum and maximum values of the analyzed variables by region.

	Schwäbische Alb	Hainich-Dün	Schorfheide-Chorin
Location	SW Germany	Central Germany	NE Germany
Size	~422 km^2^	~1300 km^2^	~1300 km^2^
Geology	Calcareous bedrock	Calcareous bedrock	Young glacial landscape
Elevation a.s.l.	460–860 m	285–550 m	3–140 m
Annual mean temperature	6–7 °C	6.5–8 °C	8–8.5 °C
Annual mean precipitation	700–1000 mm	500–800 mm	500–600 mm
N plots	158	175	309
Lichen species richness			
Mean (se)	18.61 (0.7)	5.0 (0.2)	5.9 (0.2)
Range	3–54	0–15	0–17
FDisTotal			
Mean (se)	0.3 (<0.01)	0.3 (<0.01)	0.3 (<0.01)
Range	0.2–0.5	0.2–0.5	0.2–0.5
FDisAd			
Mean (se)	0.2 (<0.01)	0.3 (<0.01)	0.3 (<0.01)
Range	0.1–0.5	0.1–0.4	0.1–0.5
FDisRe			
Mean (se)	0.3 (<0.01)	0.3 (<0.01)	0.4 (<0.01)
Range	0.2–0.4	0.2–0.5	0.2–0.5
Standing tree biomass [m^3^/ha]			
Mean (se)	323.9 (15.8)	400.1 (14.9)	441.9 (10.8)
Range	0–1017	0–882	0–1002
SMId			
Mean (se)	0.5 (<0.1)	0.4 (<0.1)	0.3 (<0.1)
Range	0–1	0–1	0–1
Deadwood cover [%]			
Mean (se)	3.7 (0.2)	3.1 (0.2)	3.8 (0.2)
Range	0.5–20.0	0.5–15.0	0.5–25.0
Rock cover [%]			
Mean (se)	1.0 (0.2)	0.2 (<0.1)	0.3 (<0.1)
Range	0–11	0–4	0–6
Proportional conifer cover			
Mean (se)	18.6 (3.2)	3.4 (1.1)	23.1 (1.9)
Range	0–100	0–91	0–100
Woody species richness			
Mean (se)	7.5 (0.3)	6.8 (0.2)	5.3 (0.2)
Range	1–16	1–14	1–16

## Data Availability

The data will be made publicly available on the BExIS platform (https://www.bexis.uni-jena.de/ (accessed on 27 January 2021)).

## Supplementary Materials

The following are available online at https://www.mdpi.com/2076-2607/9/2/463/s1. Figure S1: Effect of forest management type on lichen functional diversity for all traits (FDisTotal). Figure S2: Effect of forest management type on lichen functional diversity for ecological adaptation traits (FDisAd). Figure S3: Effect of forest management type on lichen functional diversity for reproduction, dispersal and establishment traits (FDisRe). Table S1: List of all functional traits initially compiled. Table S2: List of species and number of plots in which they were recorded, as well as functional trait values per species.
