# Supplementary material for: Direct and Indirect Effects of Management Intensity and Environmental Factors on the Functional Diversity of Lichens in Central European Forests
Source: Microorganisms. 2021 Feb 23;9(2):463. doi: 10.3390/microorganisms9020463 (PMC7926786; doi:10.3390/microorganisms9020463)
Supplement: Supplementary file 1 [file microorganisms-09-00463-s001.zip › Boch et al. Supplementary figures S1-S3.docx]

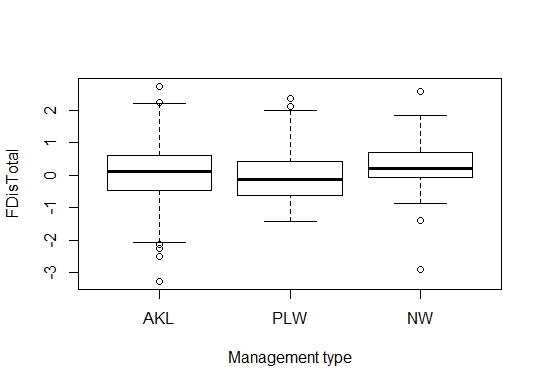


**Figure S1.** Effect of forest management type (AKL = age class forests; PLW = selection forests; NW = unmanaged forests) on lichen functional diversity of all traits (FDisTotal). F value = 1.373, d.f. = 573, p value = >0.05 (no significant differences in Tukey HSD test).


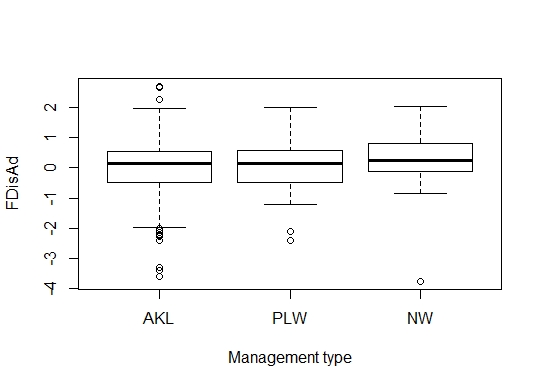


**Figure S2.** Effect of forest management type (AKL = age class forests; PLW = selection forests; NW = unmanaged forests) on lichen functional diversity associated with ecological adaptation traits (FDisAd). F value = 3.671, d.f. = 573, p-value = >0.05 (no significant differences in Tukey HSD test).


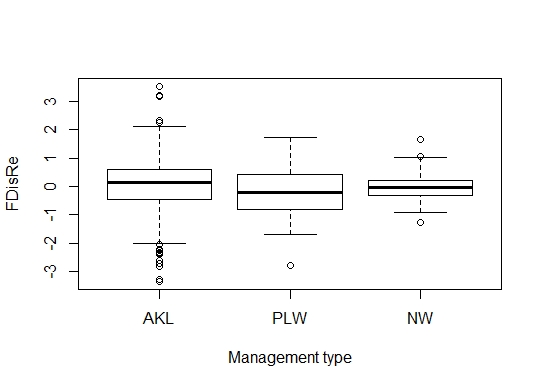


**Figure S3.** Effect of forest management type (AKL = age class forests; PLW = selection forests; NW = unmanaged forests) on lichen functional diversity associated with reproduction, dispersal and establishment traits (FDisRe). F value = 2.664, d.f. = 573, p value = >0.05 (no significant differences in Tukey HSD test).
